# Small Molecules in Parkinson’s Disease Therapy: From Dopamine Pathways to New Emerging Targets

**DOI:** 10.3390/ph17121688

**Published:** 2024-12-14

**Authors:** Hwayoung Lee, Ahmed Elkamhawy, Polina Rakhalskaya, Qili Lu, Hossam Nada, Guofeng Quan, Kyeong Lee

**Affiliations:** 1 BK21 FOUR Team and Integrated Research, Institute for Drug Development, College of Pharmacy, Dongguk University-Seoul, Goyang 10326, Republic of Korea; dong_gle1117@naver.com (H.L.); luqili220@gmail.com (Q.L.); hossam_hammouda@yahoo.com (H.N.); jeongukbong@naver.com (G.Q.); 2 Department of Chemistry, School of Sciences and Humanities, Nazarbayev University, Astana 010000, Kazakhstan; 3 Department of Biology, School of Sciences and Humanities, Nazarbayev University, Astana 010000, Kazakhstan; polina.rakhalskaya@nu.edu.kz

**Keywords:** Parkinson’s disease (PD), dopamine, small molecules

## Abstract

Parkinson’s disease (PD) is a chronic, progressive neurological disorder affecting approximately 10 million people worldwide, with prevalence expected to rise as the global population ages. It is characterized by the degeneration of dopamine-producing neurons in the substantia nigra pars compacta, leading to motor symptoms such as tremor, rigidity, bradykinesia, postural instability, and gait disturbances, as well as non-motor symptoms including olfactory disturbances, sleep disorders, and depression. Currently, no cure exists for PD, and most available therapies focus on symptom alleviation. This dopamine deficiency impairs motor control, and since dopamine itself cannot cross the blood–brain barrier (BBB), the precursor L-Dopa is commonly used in treatment. L-Dopa is administered with enzyme inhibitors to prevent premature conversion outside the brain, allowing it to cross the BBB and convert to dopamine within the central nervous system. Although these therapies have improved symptom management, recent research has revealed additional molecular factors in PD pathology, such as α-synuclein aggregation, mitochondrial dysfunction, and lysosomal abnormalities, contributing to its complexity. These discoveries open up possibilities for neuroprotective therapies that could slow disease progression. In this review, we categorize PD therapeutic targets into two main groups: currently used therapies and targets under active research. We also introduce promising small-molecule compounds studied between 2019 and 2023, which may represent future treatment options. By examining both established and emerging targets, we aim to highlight effective strategies and potential directions for future drug development in Parkinson’s disease therapy.

## 1. Introduction

Parkinson’s disease (PD) is one of the neuropsychiatric diseases suffered by about 10 million patients worldwide, and it is a disease that has attracted attention after Alzheimer’s. According to global statistics from 1996 to 2016, Parkinson’s disease deaths increased by 19.5%, and prevalence increased by 21.7% [1]. The global prevalence of Parkinson’s disease has been increasing since the 1980s, with a more pronounced rise in the past two decades, reaching 1.51 cases per 1000 people globally in 2023 [2]. The disease is generally associated with aging and has an increased risk of developing it from age 70. Men also have a 1.5 times higher chance of developing the disease than women. The year of 2017 marks 200 years since James Parkinson’s published his ‘Essay on the Shaking Palsy’ [3]. Several studies have been conducted over 200 years, but no actual treatment or drug has been developed to prevent the disease from progressing.

Symptoms of PD are usually divided into two groups: motor symptoms and non-motor symptoms. Diagnosis of Parkinson’s disease usually consists of the expression of the first motor symptom, and these symptoms gradually deteriorate with the progress of the disease with time [4,5]. There is an important difference between the motor symptoms and the non-motor symptoms. Generally, motor symptoms involve movement, while non-motor symptoms do not. There are four major motor symptoms: tremor, rigidity, bradykinesia (slow movement), and postural instability (balance problems) [6]. In the case of non-motor symptoms, it can include neuropsychiatric dysfunction, sleep disorders, autonomic dysfunction, sensory symptoms, and pain, or it can be shown as a variation in these symptoms. There are no exact directions for the development of symptoms [7]. These symptoms of Parkinson’s disease generally begin with a lack of dopamine available. The role of the dopaminergic system in neuromodulation is crucial. Dopamine is a neurotransmitter molecule that is synthesized in both central and peripheral nervous system [8,9]. The essence of its action mechanism is binding to G-protein-coupled receptors. The main function of this neurotransmitter lies in the control of body movements; however, this signaling pathway constitutes an essence in many other physiological processes. Thus, recent studies have demonstrated that dysfunctions in dopamine signaling lead to neurogenerative diseases, including Parkinson’s, Schizophrenia, Huntington’s, Attention Deficit and Hyperactivity Disorder, and Addiction. Although the exact cause of PD is not yet known, it is estimated that dopamine deficiency occurs due to the death of neurons responsible for producing dopamine [10]. Since the exact cause of PD is unknown, current treatment methods have generally been developed to maximize dopamine use. Over the last 50 years, Levodopa (L-dopa) has been a main player in Parkinson’s therapy: due to its promising effect, Parkinson’s in many patients was kept under control, allowing doctors to prevent this debilitating condition [11]. Fundamentally, L-dopa is a precursor to dopamine, a neurotransmitter in our brain, and its deficiency can lead to Parkinson’s motor symptoms. Its mechanism of action is straightforward: L-dopa crosses the blood–brain barrier and in a series of chemical reactions, is converted into dopamine in the human brain. Thus, the depleted dopamine in the corpus striatum (cluster of interconnected nuclei that make up the largest structure of the subcortical basal ganglia) is effectively restored. However, more recent studies highlighted the limitations of Levodopa: in long-term use, the development of motor fluctuations and dyskinesias across patients was recorded. As the level of dopamine in the bloodstream is constantly fluctuating, dopamine receptors in the brain are stimulated unevenly.

The stimulations are considered to occur in pulsatile, wave-like patterns, unlike the continuous dopaminergic stimulation in a healthy brain. This all accounts for the rapid progression of motor complications. In the pursuit to battle these effects, the following therapy protocol is normally administered: L-dopa is administered simultaneously with DDCI (dopa decarboxylase inhibitor), such as carbidopa or benserazide [12]. Thus, premature conversion of L-dopa to dopamine outside the brain is reversed. This approach is considered a gold standard in Parkinson’s therapy. This allows the simultaneous reduction in side effects, such as nausea and vomiting, and increases the percentage of L-dopa that reaches the brain. Some alternative protocols of L-dopa administrations were suggested as well: use of COMT inhibitors, such as entacapone. COMT is a class of catecholamine-O-methyltransferase, an enzyme family that plays a pivotal role in catecholamines metabolism [13]. Such inhibitors block the act by blocking the enzyme responsible for breaking down levodopa in the periphery, and as a result, extending the half-life of levodopa and leading to more stable and prolonged dopamine levels in the brain. As a result of entacapone uptake, the “on” time of levodopa is increased, whereas the “off” time is reduced. Several studies highlighted the improved motor symptoms in patients who uptake entacapone, often allowing for a lower daily dose of levodopa.

## 2. Dopamine Pathway

**Figure 1:**
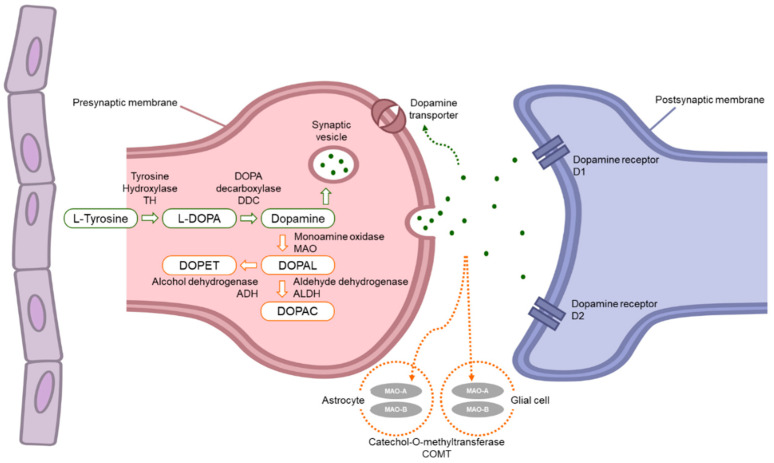
Biosynthesis and degradation pathway of dopamine.

The biosynthetic and degradation pathway of dopamine (Figure 1) is of particular interest in understanding the pathophysiology of Parkinson’s disease. Since dopamine cannot pass through the blood–brain barrier (BBB), it must be formed within the central nervous system (CNS) to be used in the striatum [14]. Mainly, the precursor for dopamine is tyrosine, but L-phenylalanine can be used in some cases. The reaction is catalyzed by an enzyme called phenylalanine hydroxylase. Overall, the synthesis is a two-step process, occurring in cytosol [10]. Firstly, tyrosine hydroxylase, a rate-limiting enzyme, converts tyrosine to levodopa (L-DOPA), using tetrahydrobiopterin, oxygen (O_2_), and iron (Fe^2+^) as a cofactor. Next, L-DOPA is converted to dopamine by aromatic l-amino acid decarboxylase (DOPA decarboxylase), and pyridoxal phosphate is a cofactor. A minor synthesis pathway may also occur, in which p-tyramine can be converted to dopamine through Cytochrome P450 2D6 activity in the substantia nigra. Following synthesis in dopaminergic neurons, dopamine is sequestered into the acidic lumen of synaptic vesicles via the vesicular monoamine transporter 2 (VMAT2). In noradrenergic and adrenergic cells, dopamine can be further converted into norepinephrine and epinephrine by sequential modifications from dopamine β-hydroxylase and phenylethanolamine N-methyltransferase in the presence of O_2_, l-ascorbic acid, and s-adenosyl-l-methionine. Lastly, the environment in the synaptic vesicle lumen is highly acidic, which allows for dopamine stabilization and oxidation prevention. It was shown that in non-acidic conditions, dopamine is sensitive to further oxidation by monoamine oxidase B (MAO-B) into 3,4-Dihydroxyphenylacetaldehyde (DOPAL) which is preferentially converted into 3, 4-dihydroxyphenylacetic acid (DOPAC) by the enzyme aldehyde dehydrogenase (ALDH) [15]. For Parkinson’s therapy, quantifying the amount of HVA (homovanillic acid) and 3-methoxytyramine is important, as these compounds are used as biomarkers for disease progression.

Dopamine metabolization goes through oxidative deamination, catalyzed by the enzyme monoamine oxidase (MAO). MAO produce 3,4-dihydroxyphenylacetaldehyde (DOPAL) from dopamine. DOPAL converts to 3,4-dihydroxyphenylacetic acid (DOPAC) by aldehyde dehydrogenase (ALDH) or to 3,4-dihydroxyphenylethanol (DOPET) by alcohol dehydrogenase (ADH). Dopamine can be metabolized to 3-methoxytyamine by the enzyme named catechol-O-methyl transferase (COMT). This 3-methoxytyamine converts to 3-methoxy-4-hydroxyacetaldehyde through MAO. At the final step, DOPAC and 3-methoxy-4-hydroxyactaldehyde turn to homovanilic acid (HVA), which is excreted with urine, through ALDH and COMT [14,16]. As the end-product, dopamine can be quantified in cerebral spinal fluid and blood. Despite this, the identification of its synthesis origin is problematic [8]. 

Since 1997, a variety of small-molecule compounds targeting Parkinson’s disease (PD) have been FDA-approved and introduced into clinical practice. Earlier therapeutic strategies for PD primarily focused on maximizing the synthesis of dopamine, a crucial neurotransmitter, and also the use of dopamine. Consequently, most current treatments involve modulating dopamine synthesis and degradation. From this perspective, the inability of dopamine to cross the blood–brain barrier (BBB) and its synthesis within the BBB by DOPA decarboxylase play an important role in designing small molecules with barrier (BBB) permeability [14]. Recent research has revealed that the motor symptoms characteristic of PD arise from the neurodegeneration of dopaminergic neurons and, in part, from the accumulation of abnormal α-synuclein protein aggregates within neurons [17]. Strategies such as gene editing and cell therapy have garnered significant attention for their efficacy in reducing toxic protein aggregate burdens in various neurodegenerative models. However, these approaches are often costly and challenging to deliver to the patient’s nervous system. Consequently, there is a pressing need for small molecules and natural products capable of lowering protein aggregation levels [18]. This review categorizes potential therapeutic targets into two groups: those currently used in therapy and those that are still under active research, including targeting α-synuclein aggregation. In the section focused on research targets, we highlight potent small-molecule compounds for PD that have been studied over the past five years, from 2019 to 2023.

## 3. FDA-Approved Therapy (Currently Available Treatments)

**Figure 2:**
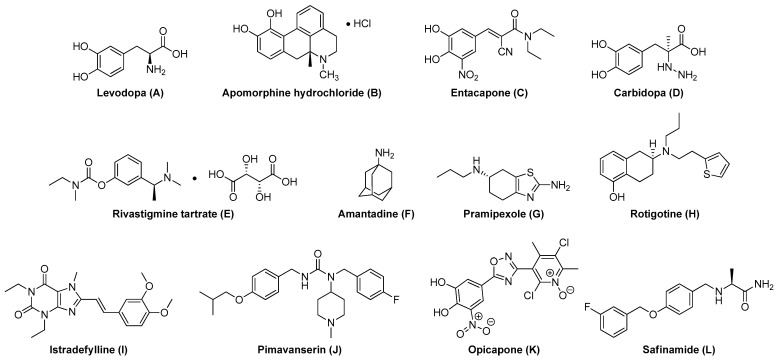
Chemical structures of FDA-approved compounds for PD.

Drugs currently given to patients with Parkinson’s disease serve as a variety of targets. (Table 1) These drugs can be classified as follows: the metabolic precursor of dopamine (Levodopa A), dopa-decarboxylase inhibitor (DDCI) (Carbidopa D), dopamine agonist (Apomorphine hydrochloride B, Amantadine F, Pramipexole G, and Rotigotine H), Catechol-O-methyltransferase (COMT) inhibitor (Entacapone C, and Opicapone K), Monoamine oxidase type B (MAO-B) inhibitor (Safinamide L), Cholinesterase inhibitor (Rivastigmine tartrate E), and Adenosine A_2A_ receptor antagonist (Istradefylline I). (Figure 2).

### 3.1. Levodopa with Dopa-Decarboxylase Inhibitor (DDCI)

Currently, the most prescribed anti-Parkinson drug is Levadopa (also known as L-dopa, dopamine precursor): it has proven efficacy in controlling the main symptoms such as slow movement. From a pathological point of view, PD is characterized by the degeneration of dopaminergic neurons in the substantia nigra of the midbrain. This condition leads to the disruption of the nigrostriatal pathway and thus decreases dopamine levels. Levodopa, a dopamine precursor, is an effective dopamine replacement agent that treats symptoms of patients who are suffering from dopamine depletion. Unlike dopamine, levodopa can cross the blood–brain barrier (BBB) [11,19].

For over 40 years, oral levodopa has been widely used for PD, but levodopa converts to dopamine in both the CNS and the periphery. To reduce its peripheral conversion to dopamine and increase central bioavailability, oral levodopa often in combination with a dopa-decarboxylase inhibitor (DDCI), such as carbidopa (D) [11,12]. However, the main limitation of levodopa is that this treatment addresses dopamine deficiency only in PD; other neurotransmitter systems are affected as well, such as serotonin, acetylcholine, and norepinephrine. In terms of the efficacy limits, several studies highlighted the development of dyskinesias (involuntary, dance-like movements, that occur after years of treatment) among the patients who were administered this drug. Overall, despite levodopa remaining a cornerstone of Parkinson’s Disease management, it may only alleviate motor symptoms in early stages but has contributed to full recovery. Recently, new-generation levodopa is almost always administered with a DDCI, like carbidopa. This combination allows for enhanced efficacy by blocking the dopa-decarboxylase functioning outside the brain: in the case of levodopa only, the administered precursor is converted to dopamine before crossing the blood–brain barrier. Despite improving the overall treatment, the side effects of levodopa, even in combination with DDCI, persist. 

### 3.2. Dopamine Agonist

As previously described, degradation of the dopaminergic neurons in the substantia nigra has been the suggested cause of Parkinson’s disease. After the remarkable result of the treatment with the dopamine precursor, levodopa, early treatment of Parkinson’s disease with dopamine precursors and DDCI has been the basis of the pharmacological treatment. At the same time, methods of increasing dopamine activity in the brain by administering dopamine receptor agonists have also emerged. Dopamine agonists are divided into two groups: ergoline and non-ergoline derivatives [20]. Unlike levodopa, they are not converted into dopamine inside the brain but rather are targeted to directly stimulate dopamine receptors and mimic the dopamine effect.

There are a large variety of dopamine agonists available. Most of the dopamine agonists used in PD are D2 dopamine receptor agonists. Ergoline derivatives (a chemical structure called ergot, that is fungus-derived), also called old dopamine agents, interact with dopamine D1 and D2 receptors as well as many other neurotransmitter receptors, such as serotonin and adrenaline receptors. New dopamine agents are non-ergoline derivatives and have a high affinity with dopamine D2 and D3 receptors. New non-ergoline agonists are a valuable option for the treatment of both early and late PD [21]. Generally, non-ergoline derivatives, such as pramipexole and ropinirole, are preferred due to the higher safety standards. 

Apomorphine hydrochloride (B) is a short-acting D1- and D2- receptor agonist and the only agent that was proven to have an equal efficacy with levodopa, though has a shorter time to onset and effect duration [22]. Amantadine (F) is known to function as a non-competitive antagonist at the phencyclidine (PCP) site within the NMDA receptor at therapeutic concentrations and to increase the release of dopamine from nerve terminals and delay its reuptake [23,24,25]. Pramipexole (G) is a selective non-ergoline dopamine agonist, which has activity at dopamine receptors belonging to the D2 receptor subfamily (D2, D3, D4 receptor subtypes) and preferential affinity to the D3 receptor subtype [26]. Rotigotine (H) can be characterized as a selective dopamine receptor agonist with a preference for the D3 receptor over D2 and D1 receptors [27]. Rotigotine, especially Neupro^®^, showed a new way for the administration of dopamine agonists with the transdermal patch (TP) that delivers the drug over 24 h period [28].

### 3.3. Catechol-O-Methyltransferase (COMT) Inhibitor

As the dopamine pathway shows, catechol-O-methyltransferase(COMT) is an enzyme that degrades neurotransmitters, dopamine, and the COMT inhibitors block this action of the COMT. These COMT inhibitors are used in combination with carbidopa-levodopa therapy to suppress motor symptoms of Parkinson’s disease [13]. Though carbidopa-levodopa therapy is the most effective to treat motor symptoms, it shows less effect for long-term treatment. By using the combination with COMT inhibitor, the effectiveness of carbidopa-levodopa therapy can be extended [29]. Regarding the main challenges in COMT clinical usage, many studies report the long-term side-effects among patients, dyskinesia being the most prominent.

### 3.4. Monoamine Oxidase Type B (MAO-B) Inhibitor

There are two isoforms of monoamine oxidase (MAO): MAO-A and MAO-B. MAO-A is the predominant isoform in the intestinal tract and plays a role in the decomposition of serotonin and catecholamines in the brain. MAO-A inhibitors are used in the treatment of psychiatric illnesses. However, their peripheral action, when applied with tyramine (present in cheese products), can induce the ‘cheese effect’, characterized by hypertension, palpitation, tachycardia, headache, and nausea [30,31].

MAO-B inhibitors are commonly used for the symptomatic treatment of PD. MAO-B is mainly distributed in platelets and glial cells, and total MAO activity in the brain is composed of about 20% MAO-A and 80% MAO-B. Both MAO-A and MAO-B control the neurotransmitters, including dopamine. MAO-A metabolizes dopamine in presynaptic neurons, while MAO-B metabolizes dopamine released to the synaptic cleft and taken up by glial cells. The number of glial cells was shown to increase with age, and in neurodegenerative diseases, as expected, the activity of MAO-B was also overexpressed [32].

**Figure 3:**
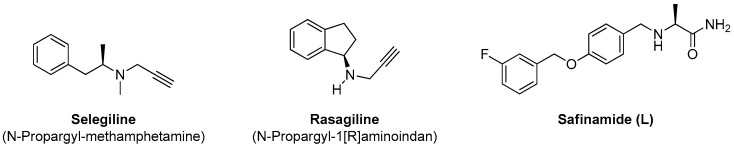
Chemical structures of MAO-B inhibitors: Selegiline, Rasagiline, and Safinamide (L).

Selegiline and rasagiline are irreversible inhibitors forming a covalent bond within the active site of MAO-B, while safinamide is a reversible MAO-B inhibitor (Figure 3). Because of irreversible inhibition of selegiline and rasagiline, these inhibitors involve unexpected side effects. Safinamide (L) is the prototype of a new generation of MAO-B inhibitors, which shows reversible inhibition [33].

### 3.5. Cholinesterase Inhibitor

Among the two categories of Parkinson’s disease symptoms, non-motor symptoms highly impact the quality of life. Especially, cognitive impairment is one of the most clinically highly related to the negative impact of non-motor symptoms [34]. Although the depletion of dopamine is the main neurochemical trait of PD, related to cognitive decline, a meaningful deficit in cortical acetylcholinesterase activity has been shown. Also, postmortem studies in patients with PD revealed that the concomitant degeneration of cholinergic and nigrostriatal pathways. Clinical trials investigating the use of cholinesterase inhibitor (ChI) treatment in PD with dementia (PDD) and dementia with Lewy bodies (DLB) showed promising results [35]. Further studies have confirmed that cognitive decline and PDD afflicts most PD patients and contributes significantly to increased morbidity and mortality [36].

An ingredient called Rivastigmine (E) was developed as a cholinesterase inhibitor developed to reduce PDD showing such a dangerous pattern and was approved by the FDA. Rivastigmine is a dual cholinesterase inhibitor, being effective on both acetylcholinesterase and butyrylcholinesterase. Various validations were conducted with the drug, and a large randomized placebo-controlled trial of 541 patients demonstrated that oral Rivastigmine (E) improved cognitive, attention and execution function, daily life activities, and behavioral symptoms six months after treatment [37].

### 3.6. Adenosine A_2A_ Receptor Antagonist

Adenosine acts as a neuromodulator, orchestrating reactions to dopamine and other neurotransmitters within brain regions involved in motor control, emotional states, and cognitive processes like learning and memory [38]. Adenosine consists of four separate receptor subtypes known as A_1_, A_2A_, A_2B_, and A_3_, all of which are part of the G protein-coupled receptor superfamily [39]. Adenosine A_1_ and A_2A_ receptors have a strong affinity for adenosine, whereas A_2B_ and A_3_ receptors display notably lower affinity for adenosine [40].

Adenosine A_2A_ receptor antagonists have been shown to effectively reverse motor deficits by binding with low doses of L-DOPA or dopamine agonists in various in vivo studies, particularly enhancing motor function without exacerbating motor impairments and preventing unintended movements. Additionally, adenosine A_2A_ receptor antagonists have shown activity in regulating non-motor symptoms of neuropsychiatric components, such as anxiety, depression, and feelings of helplessness, in animal models, suggesting their effectiveness in modulating non-motor symptoms, and also recognized as promising targets for preventing nerve loss through preclinical research [41].

**Figure 4:**
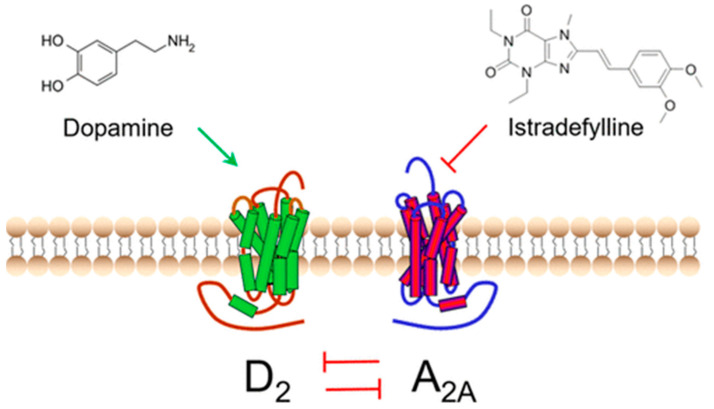
An adenosine receptor A_2A_ antagonist, Istradefylline (I) [44].

Both A_2A_ and D_2_ receptors are part of the G-protein-coupled receptor family. The A_2A_ receptor interacts with Gs protein, which promotes G-protein activity, whereas the D_2_ receptor interacts with Gi protein, which inhibits G-protein activity. A_2A_ receptors in the caudate-putamen are specifically found on spiny projection neurons, which also express D_2_ receptors on their surface, and these research results indicate that A_2A_ agonists and D_2_ agonists produce opposite effects [42]. In 2019, the FDA approved Nourianz^®^, an adenosine A_2A_ receptor antagonist developed by Kyowa Hakko Kirin Inc., Tokyo, Japan, based on clinical trial results evaluating the effectiveness of Istradefylline (I) in over 4000 PD patients, as an adjunctive treatment for levodopa during “OFF” episodes in PD (Figure 4) [43]. Unfortunately, no synthetic drugs are yet available that slow efficiently the rate of progression of Parkinson’s disease. The initial therapy, for the motor symptoms, should be constituted by direct-acting dopamine agonists while the disease progresses and these agents become insufficient, levodopa can be added. However, the efficacy of these synthetic drugs is reduced during the progression of the disease and new modalities presently under investigation are needed to improve the efficiency of clinical treatments.

## 4. Potent Targets for Parkinson’s Disease

As previously introduced, Parkinson’s disease has drugs approved by the FDA with various targets. However, as the cause of the outbreak is not properly defined, various factors that affect the disease are still being identified, and accordingly, research on small compounds targeting more factors is continuing. As already mentioned, MAO-B and Adenosine A_2A_ inhibitors are being studied in the direction of synthesizing new derivatives with more selective and good activity, and other potential Parkinson’s disease treatments targeting α-Synuclein, CK-1δ, Ca_v_ 1.3 Calcium channels, and various genetic factors are being announced. In this article, we will introduce various factors currently being studied and several potent small-molecule compounds targeting them. 

### 4.1. α-Synuclein

α-Syn, which is expressed at pre-synaptic sites in the central nervous system (CNS) at several neurotransmitter systems, was observed by Spillantini et al. to be a key component of the Lewy body, the pathological hallmark of Parkinson’s disease [45]. In terms of its structure, α-Syn is a small protein that consists of 140 amino acids with 3 distinct regions: N-terminal, which is positively charged, negatively charged C-terminal, and a central hydrophobic part, that accounts for the aggregation. Several studies confirmed that in PD patients the accumulation of insoluble cytoplasmic proteins named Lewy bodies is ubiquitous. There are two possible α-syn structures: monomer, which is predominantly observed; and tetramer, which may decompose into monomer due to intracellular denaturing agents. The exact function of α-syn and the mechanism that contributes to the death of neurons remains ambiguous, but it has been found to be involved in vesicle docking, vesicle trafficking, vesicle fusion, axonal transport, and neurotransmitter release [46]. α-syn is also found in tissues other than the CNS, such as the gastrointestinal system, which has a direct correlation to the etiology of Parkinson’s disease [47]. α-Syn has also been discovered in the epidermis and submandibular gland, and its presence in these easily accessible tissues has been proposed as a possible biomarker for Parkinson’s disease [48,49]. Intracellular aggregates of α-syn contribute to the reduced motility of neuronal vesicles and thus deteriorated protein transport. Additionally, α-syn was found to affect the lipid homeostasis inside the brain via inhibiting stearoyl- CoA-desaturase (SCD), and oleic acid-producing enzyme, reducing toxicity in α-syn overexpressing rat neurons [50]. Accordingly, the inhibition of α-Syn inhibition is a promising and novel therapeutic approach to combat Parkinson’s disease [51]. 

Under physiologic conditions, α-Syn exits in a balanced dynamic state as an oligomer [52]. α-syn has a dynamic equilibrium between soluble and membrane-bound states and is not prone to producing fibrils (highly organized, insoluble conformations identified by β-sheet conformations) [53]. Soluble α-syn monomers can form oligomers, which then join to establish small protofibrils, which aggregate into sizable, insoluble α-syn fibrils, which leads to the formation of Lewy bodies under the right conditions [54]. Due to lysosomal or ubiquitin-proteasome system’s inability to properly clear the oligomers and fibrils Lewy body pathology develops [55]. At lower pH than neutral pH, insoluble forms of α-syn are concentrated in β-sheets and are more susceptible to rapid aggregation [56]. This buildup of misfolded α-syn as β-sheets aggregates has been highly correlated with the pathogenesis of PD with the accumulation of misfolded α-synuclein in Lewy bodies and Lewy neurites representing a pathological hallmark of Parkinson’s disease [57,58]. 

**Figure 5:**
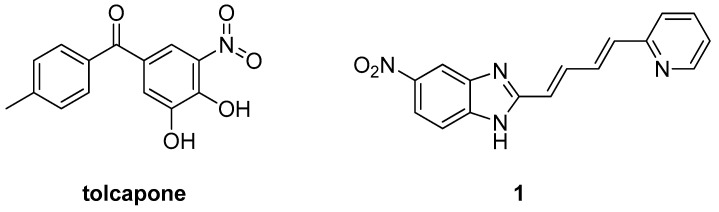
The chemical structures of tolcapone and **1** (IC_50_ = 1.09 μM).

In 2020, Longgang et al. were able to carry out high-throughput screening of small-molecule inhibitors by developing a novel series of amyloid inhibitor probes which were able to establish site-specific conjugation of aggregation-induced emission (AIE) molecules with the amyloid proteins [59]. The conventional design approach of amyloid inhibitors suffers from a shortcoming of using a specific wild-type amyloid protein to isolate a sequence-dependent amyloid inhibitor, which suffers from lowered efficiency and high error. Accordingly, Longgang et al. attempted to circumvent this shortcoming by self-assembling AIE-Aβ and AIE-αSN into amyloid-like fibrils through the typical nucleation–polymerization process which is the same process employed by their corresponding wild types. High amyloidogenic properties were maintained due to the conjugation of the probes with the two amyloid proteins, amyloid-β protein (Aβ) and α-synuclein (αSN), being Site-specific AIE conjugation (i.e., AIE@amyloid probes). Longgang et al. were able to combine the integration of AIE@amyloid probes and computational virtual screening from a large drug database to identify tolcapone as a dual inhibitor against the aggregation and cytotoxicity of both Aβ and αSN. This was achieved by molecular docking of 1742 approved small molecule drugs, obtained from the Drugbank, against αSN and Aβ and ranking those drugs based on their affinity. 100 small molecule inhibitors possessed high binding affinity to both αSN and Aβ. Out of those 100 drugs only 45 drugs were commercially available. The 45 potential candidates were further tested for their ability to inhibit the Aβ and αSN aggregation. Among the 45 candidates, only tolcapone (Figure 5) potently inhibited the Aβ and αSN aggregation, whereas tolcapone at all tested concentrations potently inhibited Aβ42 fibrillization by nearly 30–90% and αSN fibrillization by nearly 40–60% [59]. Molecular docking and simulations showed that the activity of tolcapone was due to tolcapone’s high tendency to bind and disrupt the β-sheet grooves of both Aβ and αSN assemblies, which are essential to amyloid fibrillation. Furthermore, tolcapone was demonstrated to substantially improve the spatial cognition and recognition of Aβ-treated mice [59]. This work by Longgang et al. demonstrated the high potential of combining AIE and amyloidogenic properties to rapidly and accurately identify α-Synuclein inhibitors. 

Hao et al. employed another approach to developing α-synuclein inhibitors via synthesizing a series of sheet-like conjugated compounds possessing diverse skeletons and various heteroatoms at both ends of a linker, leading to favorable π-electron delocalization and increased probability of hydrogen-bond formation [60]. The synthesized compounds with the highest activity were chosen for an IC_50_ study, which showed that the analogs possessed potent anti-aggregation activities in vitro for α-Syn with IC_50_ as low as 1.09 μM (compound **1**, Figure 5). Through investigating the IC_50_ of the effect of different substituents as well as linkers of different lengths, Hao et al. concluded that the function groups on both sides played vital roles in high activity. On the other hand, the length and type of linker did have a high impact on the inhibitory activities [60]. The chemical structure of compound **1**, the most potent derivative, is demonstrated in Figure 5. Interestingly, although the α-Syn inhibitors developed and investigated by different teams possessed different skeletons, the most potent inhibitors generally contained a NO_2_ substituent, indicating that the nitro-group plays a key role in α-Syn inhibition. The most recent studies discovered two potentially powerful α-Syn inhibitors, that may disassemble aggregated oligomers and modulate the aggregated anle138b (diphenyl-pyrazol by structure) and NPT200-11, which slows down intracellular α-Syn dimerization [61]. Both of these advanced to the phase two trials and extension studies are needed to verify clinical efficacy. 

### 4.2. CK-1δ Inhibitors

Casein kinase-1 (CK-1) plays a significant role in various neurodegenerative diseases, and its association with the phosphorylation of α-Syn has been elucidated. CK-1 is expressed in all eukaryotes, and it has been characterized by at least six isoforms (α, γ1–3, δ, and ε) in the human isoforms of CK-1 [62]. In 2017, Morales-Garcia JA et al. reported a novel isoform of CK-1, CK-1δ, demonstrating dopaminergic neuroprotection in vivo, suggesting its potential as a target for Parkinson’s disease (PD) therapy, and first reporting the efficacy of CK-1δ inhibitors as a novel candidate for disease treatment [63].

This CK-1δ has been confirmed to play various roles in subsequent sleep disorders, cancer, and various neurodegenerative diseases, including Alzheimer’s disease (AD), Amyotrophic Lateral Sclerosis (ALS), and Parkinson’s disease (PD), through years of research. Development of inhibitors in the preclinical stages has been undertaken.

**Figure 6:**
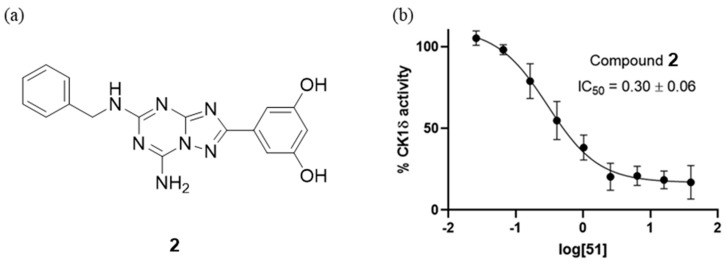
(**a**) The chemical structures of **2** (IC_50_ = 0.18 μM), (**b**) concentration–inhibition activity curve of compound **2** at FL (full-length) CK-1δ [64].

However, considering the difficulty in achieving selective inhibition of CK-1δ for the six existing human isoforms, such selectivity remains a challenging aspect for future CK-1δ inhibitor development. Grieco et al. suggested triazolo-pyrimidines (TP) and -triazines (TT) as potent scaffolds for the new class of ATP competitive CK-1δ inhibitors, and the substitutions on all positions were considered to evaluate for structure-activity relationship studies [64]. All synthesized compounds were divided into five major categories: 17 compounds of 2-substituted TP, 7 compounds of 8-substituted TP, 7 compounds of 5-substituted TP, 6 compounds of 7-substituted TP, and 20 compounds of 5-substituted TT. In total, the activity of 57 compounds was evaluated against CK-1δ. Analysis of the structure-activity relationship revealed that compound **2** (Figure 6) exhibited the best activity with an IC_50_ of 0.18 μM and demonstrated optimal results in modeling and docking studies. When evaluating the selectivity of compound **2**, it was found to primarily act on the CK1 isoform and not significantly affect other kinases, demonstrating quite good selectivity. Additionally, predictions of the blood–brain barrier (BBB) permeability for all synthesized compounds suggested that this compound scaffold could serve as a foundation for the development of CK-1δ inhibitors with improved selectivity and BBB permeability.

### 4.3. Ca_V_1.3 Calcium Channel Selective Antagonists

Ca^2+^ is essential for the process of excitation-contraction coupling in cardiac muscle cells, and L-type Ca^2+^ channels are crucial in that coupling as they facilitate the entry of calcium ions and regulate membrane excitability [65]. There are four types of L-type Ca^2+^ channels: Ca_V_1.1 and Ca_V_1.4, which are restricted to skeletal muscle and retina/immune cells, respectively, Ca_V_1.2 expressed in cardiac/smooth muscle, neurons, and endocrine cells, and Ca_V_1.3 expressed in the heart, neurons, endocrine cells, and sensory cells [66].

Substantia nigra pars compacta (SNpc) and ventral tegmental area (VTA) both contain autonomous pacemaker dopaminergic (DA) neurons, yet in SNpc, but not in VTA, the pacemaking process involves calcium influx via the L-type calcium channel, Ca_V_1.3, leading to elevated intracellular calcium levels and consequently cell death [67]. To confirm that the short isoform of Ca_V_1.3, Ca_V_1.342A, increases calcium influx, mRNA levels of Ca_V_1.342 and Ca_V_1.342A were quantified in SNpc, and their changes were tracked in the MPTP-induced Parkinson’s disease mouse model. Despite approximately 50% loss of DA neurons, mRNA levels of Ca_V_1.342 and Ca_V_1.342A remained unchanged in the SNpc following MPTP treatment. This suggests that the expression of Ca_V_1.342 and Ca_V_1.342A remains robust throughout the degenerative process in the Parkinson’s disease model [68].

Calcium channel blockers (CCBs), which are clinically used for hypertension and angina, target L-type calcium channels [69]. However, among the L-type calcium channels, Ca_V_1.2 is much more predominant than Ca_V_1.3, and CCBs inhibit both Ca_V_1.2 and Ca_V_1.3 [70]. The development of selective inhibitors for Ca_V_1.3 is needed for various disease treatments, including neuroprotection in Parkinson’s disease.

### 4.4. LRRK2 Inhibitors

Most cases of Parkinson’s disease (PD) appear to occur sporadically, but after extensive research, various genes have been identified LRRK2 genes as causative factors for this neurodegenerative disorder. Presumably, the over-activation of those is attributed to the PD progression. These genes can be classified into two categories: autosomal dominant forms and autosomal recessive forms [71]. Normally, leucine-rich repeat kinases orchestrate various cellular processes inside the cell, including vesicle transport, autophagy, and neural signal transmission [72]. Among the genetic factors classified in Autosomal dominant forms that influence Parkinson’s disease are LRRK2 [73], SNCA [74], VPS35 [75], and GBA [76]. In autosomal recessive forms, PRKN [77], PINK1 [78], and DJ-1 [79] are included. Thus, these inhibitors were confirmed to be a promising target for curing PD patients. 

Mutations in LRRK2 are implicated as the cause of 5–13% of familial PD cases and 1–5% of idiopathic PD cases [80]. Interestingly, PD mediated by LRRK2 cannot be clinically or pathologically distinguished from idiopathic PD, suggesting that studies on LRRK2 could provide clues to understanding idiopathic PD [81].

LRRK2 is a large protein consisting of several domains with different functions (2527 amino acids). LRRK2 is found to be widely expressed in many tissues such as the brain, heart, kidneys, and lungs, peripheral blood mononuclear cells (PBMCs) including lymphocytes and monocytes, dopamine neuronal areas including the cerebral cortex, cerebellum, and hippocampus, and various cellular compartments and structures associated with membranes and vesicles throughout the cytoplasm [80]. The main issue, however, lies in the difficulty of tissue-specific therapy [72].

**Figure 7:**
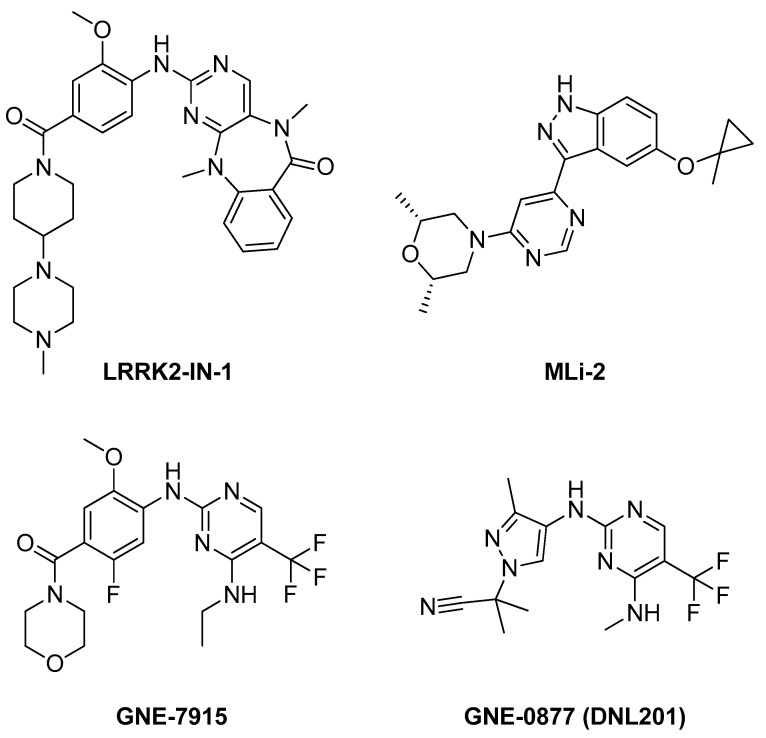
Chemical structures of previously reported LRRK2 inhibitors.

The development of inhibitors targeting LRRK2 for the treatment of Parkinson’s disease has already garnered significant attention. As a result, various compounds have been introduced as inhibitors, with some currently undergoing clinical trials. Notable among these are LRRK2-IN-1 [82], MLi-2 [83], GNE-7915 [84], and GNE-0877 (DNL201) [85,86], the structures of which are presented in Figure 7. These all differ in the action mechanism, showing the variations in the binding site. Additionally, a new inhibitor, BIIB122 (DNL151), is currently in clinical trials [87], reinforcing LRRK2 as a promising and highly regarded target.

**Figure 8:**
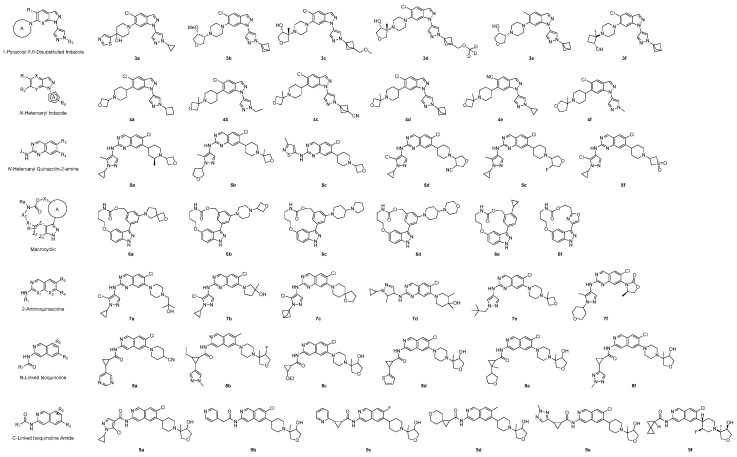
Chemical structures of six derivatives to every 7 potent scaffolds for LRRK2 inhibitor, disclosed through patents by Merck Sharp & Dohme LLC.

Merck Sharp & Dohme LLC (USA) patented 7 scaffolds for LRRK2 inhibitors between 2021 and 2023: 1-Pyrazolyl-5,6-Disubstituted Indazole [88], N-Heteroaryl Indazole [89], N-Heteroaryl Quinazolin-2-amine [90], Macrocyclic [91], 2-Aminoquinazoline [92], N-Linked Isoquinoline [93], and C-Linked Isoquinoline Amide [94]. (Figure 8). 

The IC_50_ value was determined using a GST-tagged truncated human mutant G2019S LRRK2 with the fluorescein-labeled peptide substrate LRRKtide. The LRRK2 ADP-Glo IC_50_ measurement was conducted using the ADP-Glo Kinase assay kit. Among the numerous compounds classified into seven scaffolds, representative 6 compounds with remarkable activity were selected, and their structure was disclosed. The reported compounds demonstrate strong nanomolar activity, which is detailed in Table 2. The research team of Merck Sharp & Dohme LLC provided a structural guide to design potent, brain-penetrant, and selective LRRK2 inhibitors [95]. These results present an effective structure in developing new LRRK2 inhibitors and are anticipated to significantly contribute to future research in the design of new compounds through structure-activity relationship study.

**Figure 9:**
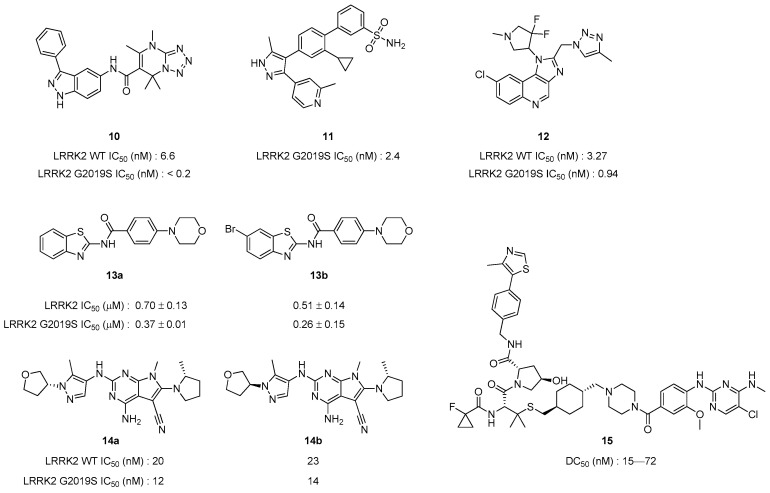
Chemical structure of new potent candidates for LRRK2 inhibitors.

Given its status as a prominent target, extensive research continues to be conducted, and here introduce newly identified compounds briefly with remarkable activity that have been reported within the last five years (Figure 9). Garofalo et al. designed and synthesized a total of 43 compounds, confirming that a few of them exhibited strong activity, with compound **10** showing the highest potency [96]. Further synthesis and analysis based on this scaffold will be instrumental in identifying promising inhibitor candidates. Leśniak R.K. et al. showed 1*H*-pyrazole biaryl sulfonamide compound **11**, which exhibited an impressive potency of 2.4 nM. The selectivity of this compound was also investigated and validated [97]. Brodney, M. A. et al. synthesized novel imidazo[4,5-C]quinolone derivatives as LRRK2 inhibitors and registered them as a patent. Among them, compound **12**, which demonstrated the most potent activity, was disclosed as the representative of this series [98]. Benzothiazole-based LRRK2 inhibitor candidates were synthesized by Zaldivar-Diez et al. and showed that Wnt/β-catenin signaling pathway could be enhanced with these compounds. Among the 21 synthesized compounds, the two most notable for their activity were designated as **13a** and **13b** [99]. Williamson et al. designed and synthesized several compounds to evaluate their activity as LRRK2 inhibitors. Notably, both compound **14a** and its diastereomer, compound **14b**, exhibited the highest potency and selectivity. These two compounds show potential as chemical probes for future LRRK2 inhibitor development studies [100]. Recently, various PROTACs have been developed and tested as protein degraders. Liu et al. developed a new PROTAC for LRRK2 degradation and synthesized compound **15**. This PROTAC compound was tested to be able to induce LRRK2 degradation, which showed significant results. This study presents a new direction for LRRK2 PROTAC development and will be an important cornerstone [101].

### 4.5. Mitochondria

Parkin and PINK1 are two genes implicated in early-stage Parkinson’s disease (PD). Individuals inheriting mutations in these genes often develop familial Parkinsonism [102]. Additionally, the observation that mitochondrial toxins can induce PD-like symptoms and that mitochondrial DNA mutations are associated with an increased risk of PD has led to the discovery of several genes linked to familial PD. The damaged mitochondria cannot be cleared efficiently, accumulating within the cell and causing further cellular damage on a molecular level. Notably, mutations in genes such as PRKN, PINK1, DJ-1, and SNCA have been shown to contribute to mitochondrial dysfunction, further highlighting the critical role of mitochondrial health in PD pathogenesis [103].

Some recent emerging targets (inosine and pioglitazone) were rejected in late stages of clinical trials as those, which have no effect on disease development. One recent advancement is NCT03840005 (ursodeoxycholic acid), which is used as an emerging target for Alzheimer’s treatment as well. Clinical trials have been conducted to evaluate the efficacy of ursodeoxycholic acid in improving mitochondrial function in Parkinson’s disease patients, aiming to develop novel therapeutic strategies for this disease, caused by mitochondrial dysfunction [104].

### 4.6. Glucocerebrosidase (GBA)

Among the European populations, the mutations in the gene encoding for glucocerebrosidase are among the most prominent causes of PD. It was established that among individuals with GBA-mutant sequences, the risk of early PD onset is 3–15 times higher, compared with a control sample [61,105]. The FDA-approved therapy for such Patients is a glucocerebrosidase gene therapy (PR001), that is administered to both PD patients and those affected with Gaucher’s disease—the promising results were obtained. One more emerging target in preventing PD symptoms is ambroxol, the chaperone of the lysosomal enzyme glucocerebrosidase. In vitro and in vivo studies have demonstrated that ambroxol enhances β-glucocerebrosidase enzyme activity and decreases α-synuclein levels [106,107,108]. Currently, this drug is under phase 3 clinical trials.

## 5. Conclusions

Parkinson’s disease is a heterogeneous condition that many patients suffer from, with diverse pharmacologic approaches applied. Numerous drugs have been developed and approved by the FDA to alleviate the symptoms of Parkinson’s disease, including various drugs that affect the dopamine biosynthesis pathway, including L-DOPA, a precursor to dopamine. These drugs contribute to reducing patients’ suffering by increasing the amount of dopamine produced and used, thereby regulating motor and non-motor symptoms. However, they do not ultimately prevent or eliminate the fundamental dopamine deficiency underlying the disease or its progression. This necessitates various mechanistic studies and the synthesis of new drugs based on the researched mechanisms. Conducting research on the various mechanisms provided in this review could lead to the development of more effective new drugs or the discovery of the fundamental causes of Parkinson’s disease through the analysis and cross-referencing of mechanisms. The provided structures not only serve as promising lead compounds for their respective targets but also hold potential as biomarkers to investigate the roles of these targets in Parkinson’s disease. Although existing pharmacotherapies have not achieved a definitive cure, the development of novel small-molecule therapeutics targeting newly identified disease mechanisms offers hope for achieving the goal of a radical cure. Despite not currently offering therapies to prevent or delay disease onset, proper pharmacological treatment may significantly decrease PD adverse effects. Some further studies and investigations are required, that could lead to significant advancements not only in the treatment strategies for Parkinson’s disease but also for various nondegenerative and psychiatric disorders. In the future, the therapeutic landscape of Parkinson’s is predicted to rely not solely on symptoms-modification, but rather on disease-modifying therapies.

## Figures and Tables

**Table 1:** The list of drugs approved by the Food and Drug Administration (FDA) for sale in the United States.

	Drug Classification		Drug Name	Ingredient	Year
1	Levadopa and derivatices		Duopa enteral suspension	Levodopa (A) and Carbidopa (D)	2015
			Rytary		2015
2	Non-ergoline dopamine agonist		Mirapex	Pramipexole (G)	1997
			Apokyn	Apomorphine hydrochloride (B)	2004
			Kynmobi		2020
			Neupro	Rotigotine (H)	2012
3	Catechol-O-methyltransferase (COMT) inhibitor		Comtan	Entacapone (C)	1999
			Ongentys	Opicapone (K)	2020
4	Monoamine oxidase type B (MAO-B) inhibitor		Xadago	Safinamide (L)	2017
5	Ohters	Cholinesterase inhibitor	Exelon Patch	Rivastigmine tartrate (E)	2007
		Dopamine agonist, NMDA receptor antagonist	Gocovri	Amantadine (F)	2017
		Adenosine A_2A_ receptor antagonist	Nourianz	Istradefylline (I)	2019
		Atypical antipsychotic	Nuplazid	Pimavanserin (J)	2016

**Table 2:** Biological activity of 42 compounds tested for LRRK2 inhibition.

**1-Pyrazolyl-5,6-Disubstituted Indazole**	**LRRK2 IC_50_ (** **μM)**		**N-Heteroaryl Indazole**	**LRRK2 IC_50_ (** **nM)**	
	**3a**	0.09751		**4a**	0.90
	**3b**	0.1043		**4b**	0.80
	**3c**	<0.0804		**4c**	<0.625
	**3d**	0.1570		**4d**	0.71
	**3e**	0.2592		**4e**	<0.625
	**3f**	0.1540		**4f**	<0.625
**N-Heteroaryl Quinazolin-2-amine**	**LRRK2 pIC_50_** *		Macrocyclic	**LRRK2 ADP-Glo** **IC_50_ (nM)**	
	**5a**	>9.20		**6a**	<10
	**5b**	>9.20		**6b**	<10
	**5c**	>9.20		**6c**	<10
	**5d**	>9.20		**6d**	<10
	**5e**	>9.20		**6e**	<10
	**5f**	>9.20		**6f**	<10
**2-Aminoquinazoline**	**LRRK2 pIC_50_ (nM)** *		N-Linked Isoquinoline	**LRRK2 pIC_50_ (nM)** *	
	**7a**	10.12		**8a**	10.09
	**7b**	9.772		**8b**	10.09
	**7c**	9.759		**8c**	10.08
	**7d**	10.19		**8d**	10.09
	**7e**	9.727		**8e**	10.00
	**7f**	9.346		**8f**	10.09
**C-Linked Isoquinoline Amide**	**LRRK2 pIC_50_ (nM)** *		*pIC_50_ = −log_10_(IC_50_)		
	**9a**	10.09			
	**9b**	10.09			
	**9c**	10.09			
	**9d**	10.09			
	**9e**	10.09			
	**9f**	10.09			
